# Efficient production of functional cholera toxin B subunit using geminiviral replicons in *Nicotiana benthamiana*

**DOI:** 10.3389/fbioe.2025.1693569

**Published:** 2025-11-14

**Authors:** Nan-Sun Kim, Jihyea Lee, Juho Lee, Seon-Kyeong Lee, Kyeong-Ryeol Lee

**Affiliations:** Department of Agricultural Biology, National Institute of Agricultural Sciences, Rural Development Administration, Wanju, Republic of Korea

**Keywords:** cholera toxin B, geminivirus-based deconstructed vectors, transient expression, *Nicotiana benthamiana*, ER stress

## Abstract

The cholera toxin B subunit (CTB) has the potential to be a carrier molecule and an effective adjuvant for mucosal vaccines because of its ability to enhance immune responses to antigens. CTB proteins have been expressed in plant-based expression systems. In this study, we used geminiviral replicon systems to transiently express CTB in *Nicotiana benthamiana*. We developed a high-level expression system that uses combinations of the replication machinery of geminivirus, including tomato yellow leaf curl virus (TYLCV), honeysuckle yellow vein virus (HYVV), and beet mild curly top virus (BMCTV). These were named TIR + TC123, HIR + HC123, and BIR + BC1, respectively. The plant-optimized *CTB* gene was cloned into each geminivirus IR-carrying vector and co-infiltrated into *N. benthamiana* leaves. Immunoblot analysis verified the synthesis and assembly of CTB into pentamers. The highest CTB protein level, approximately 2.5 mg/g fresh weight (22% of total soluble protein), was observed on day 5 in the BMCTV combination in *N. benthamiana*. CTB transiently expressed in plants using geminivirus-based viral vector systems demonstrated enhanced protein expression levels and a strong affinity for GM_1_-ganglioside. This suggests that the CTB subunits form an active pentamer, implying its potential as an adjuvant for mucosal vaccines.

## 1 Introduction

Recent advances in plant viral vectors, particularly those using deconstructed vectors delivered to *Nicotiana benthamiana*, have made it possible to rapidly and affordably produce high yields of recombinant proteins, such as vaccines and monoclonal antibodies (Eidenberger et al., 2023; Akher et al., 2025). This approach overcomes the limitations of full-virus systems, such as restrictions on foreign gene size and the potential for biohazards, by eliminating undesirable viral genes and functions (Gleba et al., 2004; Chen et al., 2011). Efficient transient expression vectors have been developed using the modified genomes of both RNA and DNA viruses. Among RNA virus-based vectors, several strategies have been used to achieve high expression, including well-known deconstructed vectors, such as magnICON (Marillonnet et al., 2005; Gleba et al., 2007), TRBO (Lindbo, 2007), and pEff (Mardanova et al., 2017), as well as non-replicating vectors, such as pEAQ (Peyret et al., 2019; Castells-Graells and Lomonossoff, 2021) and pTRA (Maclean et al., 2007). These RNA viral vectors have been optimized for economic mass production, with industrial process designs focused on cost-effective yields and rapid scale-up (Gleba et al., 2014; Abrahamian et al., 2020; Schillberg and Finnern, 2021). The DNA geminivirus replicon system, bean yellow dwarf virus (BeYDV), was designed using module combination, resulting in multiple viral replicons. The BeYDV system can express multiple genes simultaneously and manage large gene fragments across a variety of dicot plants (Diamos et al., 2016; Diamos and Mason, 2018; Diamos et al., 2020a). This feature makes BeYDV an excellent tool for recent research, such as gene editing and synthetic biology applications (Gong et al., 2024; Garcia-Perez et al., 2025).

Our previous research focused on three geminivirus-derived deconstructed DNA vector systems to express turbo GFP (tGFP) in *N. benthamiana* leaves (Kim et al., 2024). We found that combinations involving the intergenic region (IR) and replication-related gene (C123) from both tomato yellow leaf curl virus (TYLCV) and honeysuckle yellow vein virus (HYVV) produced the highest tGFP yields. When co-expressed with the post-transcriptional gene silencing (PTGS) suppressor p19, tGFP production reached an impressive 1.1–1.2 mg/g fresh weight (FW) and 10.6%–12.1% of the total soluble protein (TSP) in the HYVV- and TYLCV-derived C123 systems, respectively. Conversely, the beet mild curly top virus (BMCTV) combination did not yield comparable maximum production levels. These findings highlight the need for continued research and experimentation with various target proteins to further optimize recombinant protein production in geminiviral vector systems.

The cholera toxin produced by *Vibrio cholerae* is a multifunctional entity composed of a single active A subunit and five identical B subunits (CTB). The A subunit’s two domains, A1 and A2, are responsible for catalysis and anchoring to the CTB pentamer. This CTB pentamer, which comprises five identical polypeptides, targets glycosphingolipid receptors on eukaryotic cell surfaces. Although CTB does not possess enzymatic function, its role in enabling the toxin to bind to the host cell membrane is indispensable (Guatrecassas, 1973; Holmgren, 1981). The non-toxic CTB has been reported as a representative adjuvant that efficiently induces mucosal immunity and could provide a safe alternative to evaluate these toxins as mucosal adjuvants in humans and animals (McGhee et al., 1992). Thus, these properties of CTB, various expression platforms have been explored for the expression of recombinant CTB and its fusion proteins, including prokaryotic hosts like engineered *Escherichia coli* (Gong et al., 2009; Zeighami et al., 2010; Khastar et al., 2022), and *Lactobacillus* species (Okuno et al., 2013; Hiramatsu et al., 2014), as well as eukaryotic systems such as yeast (Arzanlou et al., 2005), silkworms (Li et al., 2014), and plants (Arakawa et al., 1997; Jani et al., 2002). Although plant-derived CTB expressed in plants has the same antigenic determinants as native CTB, its low expression levels restrict the advancement of effective plant-based vaccines. To enhance CTB expression, researchers have reported success by either appending an endoplasmic reticulum (ER) retention signal or optimizing the prokaryote-derived sequences of CTB into plant-compatible versions, leading to increased stable mRNA and protein expression (Rattanapisit et al., 2013; Kajiura et al., 2013; Morris et al., 2021).

Ultimately, in plant molecular farming, strategies for producing high-quality proteins, such as therapeutics and vaccines, involve either secreting the target protein or accumulating it in the ER using an ER-retention signal (Eidenberger et al., 2023). The ER is essential for proper protein development because its lumen is rich in molecular chaperones and enzymes that facilitate the correct folding and assembly of proteins. The ER Quality Control (ERQC) system monitors proteins for correct folding, and any improperly folded proteins are marked for destruction through ER-associated degradation (ERAD) (Howell, 2013). However, overproduction of proteins via transient expression in plants or unfavorable environmental conditions can lead to the accumulation of misfolded proteins and induce ER stress. To cope with this, cells activate the unfolded protein response (UPR), boosting the expression of protein-folding genes. Once activated, the UPR restores cell homeostasis using complementary mechanisms to reduce translation and boost ERQC and ERAD capabilities. If ER stress is severe, prolonged UPR activation can lead to programmed cell death, a mechanism that protects stressed tissues from dysfunction (Kørner et al., 2015). Therefore, understanding ER stress induced by transient protein overexpression in plants is important in plant-derived recombinant protein production systems. Plant-based transient expression systems leveraging geminivirus replication machinery have emerged, enabling a substantial increase in the copy number of replicons containing transgenes within the plant nucleus and, consequently, enhanced target gene transcription in *N. benthamiana* (Huang et al., 2009; 2010). However, a significant challenge with these geminiviral vector systems, particularly BeYDV vectors, is the frequent occurrence of severe tissue necrosis when expressing certain proteins, including the Ebolavirus glycoprotein, hepatitis B core antigen, GII norovirus particles, monoclonal antibodies, and other ER-targeted proteins (Phoolcharoen et al., 2011; Mathew et al., 2014; Diamos and Mason, 2019). Although geminiviruses have evolved sophisticated counter-defense mechanisms, the continuous interplay between viral virulence and host immunity can still lead to cellular damage and death, especially when the viral load or host recognition reaches a critical threshold. Therefore, a thorough understanding of these factors is crucial for optimizing geminiviral vector systems for diverse applications, from biopharmaceutical protein production to gene editing, by minimizing undesirable cell death and maximizing desired outcomes.

In this study, we investigated the high-level expression of recombinant CTB using Agrobacterium-mediated delivery of three geminivirus-based deconstructed vectors in *N. benthamiana*. We designed and constructed a synthetic *CTB* gene optimized for enhanced expression in plants by incorporating ER-retention sequences. The BMCTV combination yielded the highest CTB protein, which was confirmed to be biologically functional. This plant-produced CTB formed active pentamers with a strong binding affinity for GM_1_-ganglioside, similar to commercially available CTB, suggesting its potential as an adjuvant for mucosal vaccines.

## 2 Materials and methods

### 2.1 Vector construction

**FIGURE 1:**
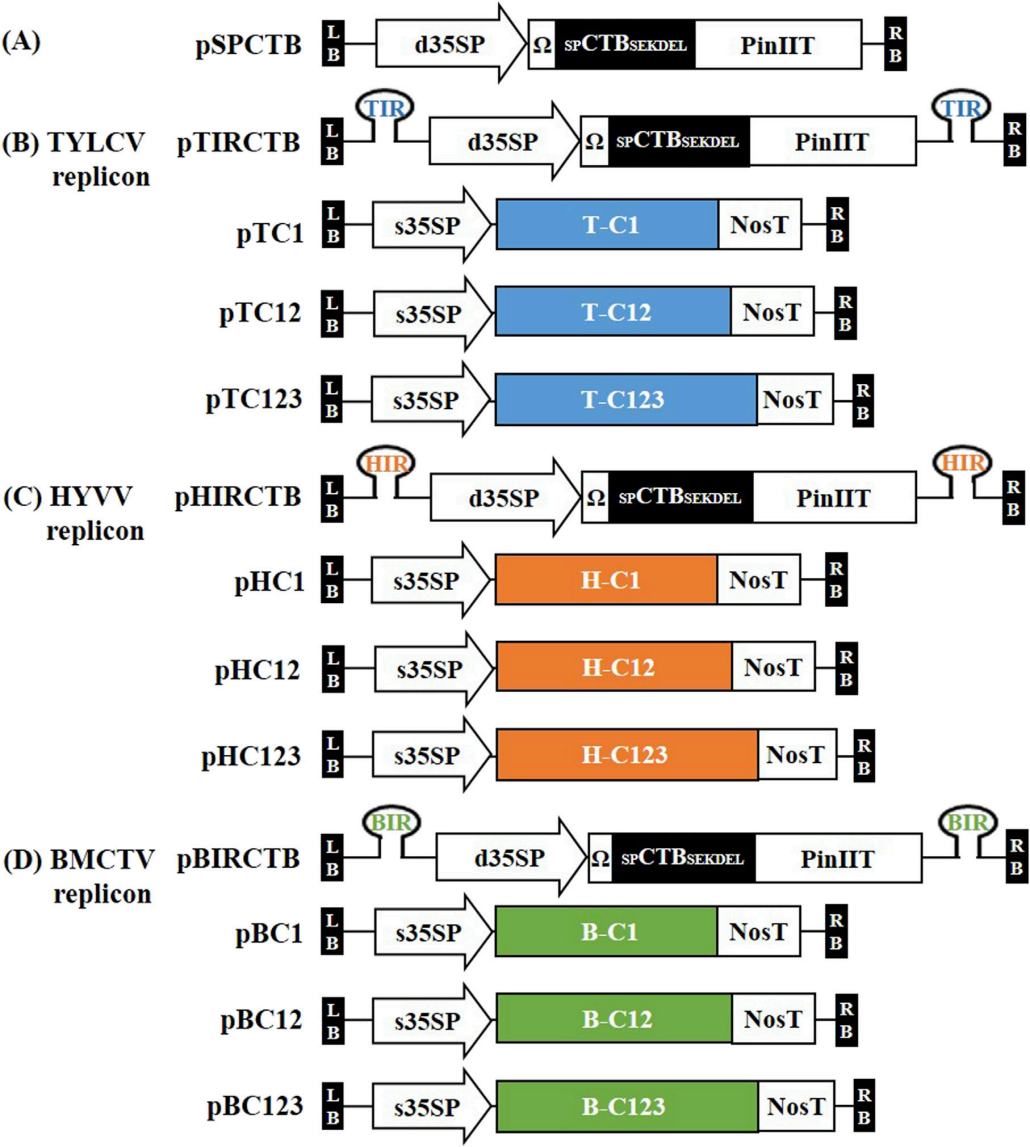
Schematic representation of the plant expression vector used in this study. **(A)** Plasmid pSPCTB (non-replicating control), containing codon-optimized CTB fused with its own signal peptide at the N-terminal end and SEKDEL endoplasmic reticulum (ER) retention signal at the C-terminal end, was synthesized and introduced into the plant expression vector under the CaMV 35S double promoter (d35SP) with a 5ʹ-leader sequence of tobacco mosaic virus (Ω) and potato protease inhibitor II terminator (PinIIT). **(B–D)** The intergenic region (IR) of TYLCV, HYVV, and BMCTV are located in pSPCTB inside the T-DNA region with a hairpin structure cloned into the Level M vector and named pTIRCTB, pHIRCTB, and pBIRCTB, respectively. To form the viral replicons, replication initiation proteins were supplied by open reading frames (ORFs) from TYLCV, HYVV, or BMCTV. These ORFs–C1 (C1/ΔC2; with a modified C2 start codon), C12 ((C1/C2/ΔC3; with a modified C3 start codon), and C123 ((C1/C2/C3)–were incorporated into previously developed vectors (Kim et al., 2024). The resulting vectors were named as follows: pTC1, pTC12, and pTC123 containing TYLCV ORFs; pHC1, pHC12, and pHC123 containing HYVV ORFs; and pBC1, pBC12, and pBC123 containing BMCTV ORFs. All ORFs were expressed under the CaMV 35S short promoter (s35SP) and nopaline synthase terminator (NosT). LB and RB indicate the T-DNA left and right borders, respectively.

Previously developed three types of geminiviral-derived deconstructed vector replicon systems (Kim et al., 2024) were used for *CTB* expression in *N. benthamiana*. For the ER-targeting expression vector, the nucleotide sequence of *CTB* (GenBank Accession No. MG356518) harbored a native signal sequence linked with SEKDEL at the C-terminus, which was plant-codon-optimized, synthesized, and cloned into the pBHK cloning vector (Bioneer, Daejeon, Republic of Korea). *CTB* (L0, pBHK), cauliflower mosaic virus (CaMV) double 35S promoter (d35SP), tobacco mosaic virus (TMV) 5ʹ-leader sequence (Ω) (L0; pICH51288), and potato protease inhibitor II terminator (PinIIT) (L0; pBHK) were inserted into the Level 1–2 destination vector (L1-2; pICH47751) using the Golden Gate cloning method with *Bsa*I to construct the *CTB* expression vector pSPCTB. Each IR was cloned into L1-1 and L1-3 using polymerase chain reaction (PCR) with specific primers (Supplementary Table S1). Each IR (in L1-1 and L1-3), pSPCTB (L1-2), and end linker L3E were inserted into the Level M1 destination vector (pAGM8031) using the Golden Gate cloning method with *Bpi*I to construct IR-carrying CTB expression vectors, that is, TYLCV-based IR vector, pTIRCTB; HYVV-based IR vector, pHIRCTB; and BMCTV-based IR vector, pBIRCTB. The pSPCTB expression vector was used as a non-replicating control. A schematic representation of the geminivirus-based deconstructed vectors is shown in Figure 1.

### 2.2 *Agrobacterium tumefaciens*-mediated transient expression

Binary vectors were introduced separately into *Agrobacterium tumefaciens* GV3101 using 50–500 ng of recombinant plasmids via the freeze-thaw method, as described by Jyothishwaran et al. (2007). Recombinant strains were grown overnight at 28 °C with agitation in LB medium supplemented with the appropriate antibiotics for infiltration. Cells were pelleted by centrifugation at 2,000 × g for 5 min, suspended in infiltration buffer [10 mM 2-(N-morpholino) ethanesulfonic acid (MES) (pH 5.6) and 10 mM MgCl_2_ supplemented with 100 μM acetosyringone], and diluted in infiltration buffer to OD_600_ = 0.5, unless otherwise described. When mixing the two constructs, each Agrobacterium concentration was set to OD_600_ = 1.0 and mixed 1:1. For co-infiltration with tomato bushy stunt virus (TBSV) p19, each was set to OD_600_ = 1.0, and mixed 1:1:0.5. After incubation at 25 °C for 3 h, cells were infiltrated into the leaves of 4–5 weeks old *N. benthamiana* plants grown in a hydroponic growing medium at 23 °C under a 16 h light and 8 h dark photoperiod at a light intensity of 100 mol m^-2^ s^-1^. The resulting bacterial suspensions were infiltrated into fully expanded leaves using a syringe without needles through a small puncture (Huang et al., 2006).

### 2.3 Polymerase chain reaction (PCR) analysis

Genomic DNA was isolated from plant tissue using NucleoSpin Plant II (Machery-Nagel GmbH and Co, Düren, Germany) according to the manufacturer’s instructions. Genomic DNA was quantified using a NanoDrop spectrophotometer (Thermo Fisher Scientific, Waltham, MA, USA) and diluted to 50 ng/μL. PCR was performed in reaction volumes of 20 μL using 100 ng of genomic DNA and Ex-Taq DNA polymerase (Takara, Japan). The primers (Supplementary Table S1) used in the PCR analysis were designed to amplify each replicon recirculation and C1, C12, and C123 in the co-infiltrated *N. benthamiana* genome. The PCR products were electrophoresed on 1.0% (w/v) agarose gels, stained with StaySafe Nucleic Acid Gel Stain (Real Biotech Corporation, Taiwan), and visualized under ultraviolet light.

Quantitative PCR was performed using a CFX96 Touch Real-Time PCR Detection System (Bio-Rad, Hercules, CA, USA) real-time PCR machine together with AccuPower 2× GreenStar qPCR Master Mix (Bioneer, Republic of Korea). Triplicate reactions were performed, and the tGFP gene copy numbers were normalized using *β-actin* of *N. benthamiana* (GenBank accession No. JQ256516) was used as a reference. The PCR primer sequences are listed in Supplementary Table S1.

### 2.4 Quantitative reverse transcription PCR (qRT-PCR)

Total RNAs was purified from infiltrated *N. benthamiana* leaves at 1, 2, 3, 5, and 7 days post-infiltration (DPI) using the Spectrum™ Plant Total RNA Kit (Sigma-Aldrich, St. Louis, MO, USA), and the residual DNA was removed with RNase-free DNase I (Takara). First-strand cDNA was synthesized from 5 μg of total RNA and oligo dT primers using the RNA-to-cDNA EcoDry Premix (Takara) according to the manufacturer’s protocol. Quantitative reverse transcription PCR analysis was performed using 100 ng of cDNA in a 20 μL reaction volume using AccuPower 2× GreenStar™ qPCR Master Mix (Bioneer). The gene-specific primers used are listed in Supplementary Tables S1, S2, qRT-PCR was performed with an initial step at 95 °C for 10 min, followed by 40 cycles of 95 °C for 20 s, 58 °C for 20 s, and 72 °C for 25 s. Fluorescence was recorded after the last step of each cycle. Three replicates were performed for each sample. Amplification, data processing, and detection were performed using a CFX96 Real-Time PCR Detection System (Bio-Rad). Quantification cycle (Cq) values were examined using the 2^−ΔCT^ method to determine the changes in gene expression.

### 2.5 Biophysical assays

After agroinfiltration, leaves were collected at 1, 2, 3, 5, and 7 DPI. Portions of the collected leaves were cut, weighed to determine FW, and subjected to CTB and TSP analyses. The remaining leaves were weighed and subsequently oven-dried at 60 °C for 3 days to determine the dry weight (DW) and dry matter content (DMC; ratio of DW to FW). The leaves were homogenized in a buffer mixture, and the homogenate was centrifuged as previously described (Pruksarojanakul et al., 2025). For biological replicates, three plants were prepared per DPI, temperature, and expression vector for both experiments.

### 2.6 Protein extraction

TSPs were extracted by homogenizing agroinfiltrated leaf samples harvested 1, 2, 3, 5, and 7 DPI with 1:2 (w/v) ice-cold extraction buffer [200 mM Tris-Cl (pH 7.0), 100 mM NaCl, 10 mM EDTA, 0.5% Triton X-100, and a protease inhibitor cocktail (Roche Diagnostics GmbH, Mannheim, Germany)]. Cleared supernatants were obtained by centrifugation at 15,000 × *g* for 15 min at 4 °C. The protein concentration in the leaf samples was determined using the Bradford Protein Assay Reagent kit (Bio-Rad) (Bradford, 1976), with bovine serum albumin (BSA) as the reference standard.

### 2.7 Western blot analysis

The expression of CTB in *N. benthamiana* was analyzed using Western blotting. Briefly, TSP (25 μg) was mixed with sample buffer [10% glycerol, 60 mM Tris–HCl (pH 6.8), 2% SDS, 0.5 M dithiothreitol, 0.01% bromophenol blue] at 25 °C and separated by 12% SDS-polyacrylamide gel electrophoresis. The proteins were then electrophoretically transferred to an iBlot 2 polyvinylidene fluoride (PVDF) Regular Stacks (Invitrogen, Carlsbad, CA, USA) according to the manufacturer’s instructions. The protein-transferred membranes were blocked with 5% non-fat skim milk in Tris-buffered saline (TBS) containing 0.05% Tween-20 (TBST, pH 7.4) for 2 h at 25 °C. CTB protein was detected using a mouse anti-CT polyclonal antibody (Sigma) at 1:5,000 dilutions. The secondary antibody used was alkaline phosphatase-conjugated goat anti-mouse IgG (Sigma) at 1:10,000 dilutions. The membranes were developed using nitro blue tetrazolium chloride and 5-bromo-4-chloro-3-indolyl phosphate (Sigma). Purified HEK 293 cells expressing CTB protein (Sigma) were used as a positive control.

### 2.8 Enzyme-linked immunosorbent assay (ELISA)

The CTB protein levels extracted from agroinfiltrated leaf tissues were determined using indirect ELISA. Briefly, a 96-well Maxisorp microtiter plate (NUNC, Roskilde, Sjelland, Denmark) was coated with CTB plant extracts in coating buffer (15 mM Na_2_CO_3_, 35 mM NaHCO_3_, pH 9.6) at 4 °C overnight, and washed four times with 200 μL of phosphate-buffered saline (PBS) with 0.1% Tween-20 (PBST, pH 7.4). The wells were then blocked with 1% BSA in PBS at 37 °C for 2 h. After washing the wells three times, 100 μL of diluted anti-rabbit CTB polyclonal antibody (1:5,000) (Sigma) was added to the wells and incubated at 37 °C for 2 h. After washing four times, anti-rabbit IgG-conjugated alkaline phosphatase (1:10,000) (GenDepot, Baker, TX, USA) was added to each well and incubated at 37 °C for 2 h. After five washes, 100 μL of 3,3′,5,5′-tetramethylbenzidine (TMB) containing H_2_O_2_ solution was added, and the plate was incubated for 15 min at 25 °C. The enzymatic reaction was stopped by quickly adding 50 μL of 2 N H_3_PO_4_ to each well. Absorbance at 450 nm was measured using a microplate reader. To calculate the relative amount of CTB in the plant sample, the OD value from each sample was subtracted from the untransformed plant OD value before conversion using an ELISA standard curve constructed with purified CTB expressed in HEK 293 cells (Sigma). Plant samples were analyzed by dilution from 1:1,000 to 1:5,000 with a coating buffer.

### 2.9 GM_1_-ELISA

The GM_1_–ELISA assay was performed to determine the binding capacity of plant-derived CTB protein to GM_1_-ganglioside. Briefly, the microtiter plate was coated with GM_1_-ganglioside (Cayman Chemical, Ann Arbor, USA), 3 μg/mL dissolved in bicarbonate buffer (15 mM Na_2_CO_3_, 35 mM NaHCO_3_), pH 9.6 (100 μL per well) at 4 °C overnight. BSA-coated wells were used as controls. All wells were blocked with 200 μL of 3% fat-free milk in PBS and incubated at room temperature for 2 h. After washing with PBST, 100 μL of TSP extracts from transgenic or wild type plants were added to the wells coated with GM_1_-ganglioside or BSA. The wells added with HEK 293 cell-derived CTB (Sigma) were used as a positive control. The plates were incubated overnight at 4 °C. After washing, the binding of plant-derived CTB to GM_1_-ganglioside was visualized by the addition of 100 μL of rabbit anti-cholera toxin antibody (Sigma) diluted 1:5,000 in PBS containing 1% BSA for 1 h at 37 °C, followed by the addition of 100 μL of enzyme-conjugated anti-rabbit IgG and enzyme substrate as for a conventional ELISA, as described above. Horseradish peroxidase-conjugated antibodies were diluted 1:10,000 before use in the assay. As the substrate, 100 μL TMB peroxidase substrate (Sigma) was used as the substrate. After 10 min of incubation at room temperature, the OD at 450 nm was measured as described above. Commercial CTB (Sigma) was used as a positive control in the GM_1_ ELISA.

### 2.10 Statistical analyses

Data were analyzed using an analysis of variance (ANOVA) to evaluate the expression levels of the target genes and proteins, and dry matter content. One-way ANOVA, followed by Dunnett’s post-hoc test or Tukey’s honestly significant difference test, was used to compare the means of the treatments in each experiment. Statistical analyzed were conducted using Graph Pad Prism 10.4.2.

## 3 Results

### 3.1 Efficient geminiviral system-based expression of *CTB* in *Nicotiana benthamiana* leaves

In a previous study, we reported the industrialization potential by confirming high tGFP expression using geminivirus-derived deconstructed vector combination systems (Kim et al., 2024). Here, we cloned the vectors (Figure 1) to confirm the high expression of the CTB protein using the developed geminivirus-based deconstructed vector systems. For *CTB* expression, the *CTB* codon was optimized by *the Nicotiana tobacum* codon and cloned into each geminivirus-based deconstructed vector using MoClo systems with each IR and replication-related gene for the amplification of episomal replicons and high protein expression. The IR-carrying CTB vector, pTIRCTB, pHIRCTB, and pBIRCTB, was controlled by d35SP, Ω, and PinIIT, respectively. pSPCTB was used as a negative control (Figure 1). To identify the best combination for high CTB expression, we used Rep-supplying vectors (C1ΔC2, C1/C2/ΔC3, and C1/C2/C3, named pTC1/pHC1/pBC1, pTC12/pHC12/pBC12, and pTC123/pHC123/pBC123, respectively) that were developed previously and whose expression was regulated by the CaMV short 35S promoter (s35SP) and *Agrobacterium tumefaciens* nopaline synthase terminator (NosT) (Kim et al., 2024). Agrobacterium strains carrying these constructs were co-infiltrated at the same ratio, and *N. benthamiana* leaves were sampled in a time-dependent manner.

### 3.2 Correlation of *CTB* copy number, mRNA, and protein production in *Nicotiana benthamiana*

Genomic DNA was extracted from leaf samples collected at 3 DPI (Regnard et al., 2010) to confirm the formation of episomal replicons containing three IRs in co-infiltrated leaves. The formation of episomal replicons from T-DNA is essential for amplification using primers oriented at both ends (Supplementary Figure 1A, B). Approximately 1.2 kb PCR products were amplified only from circularized unit-length replicons. The results showed that replicon formation occurred in co-infiltrated *N. benthamiana* leaves.

**FIGURE 2:**
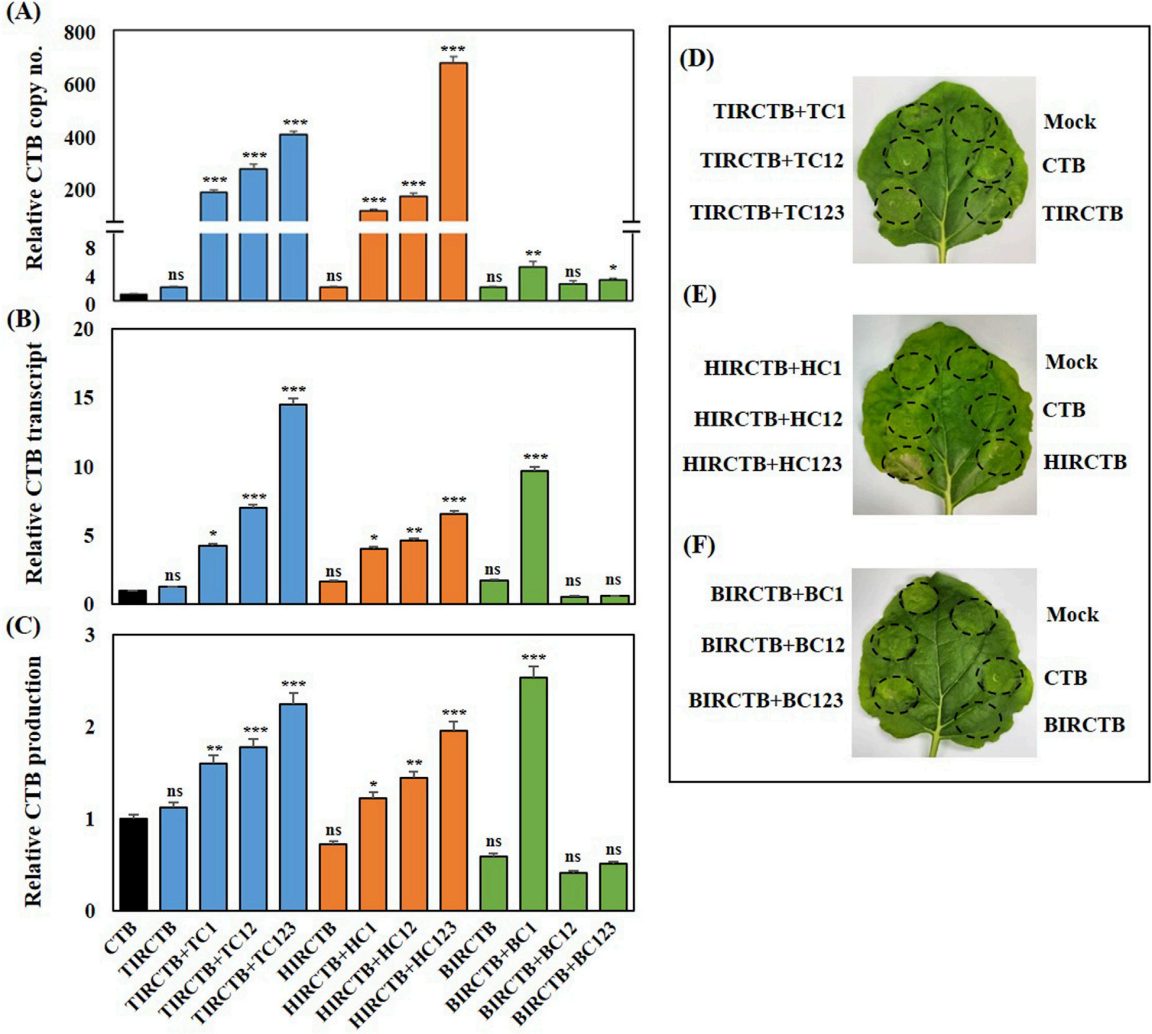
Transient expression of CTB in agroinfiltrated *Nicotiana benthamiana* leaves at 3 DPI. **(A)** Relative copy number analyzed via qRT-PCR using primers specific for *CTB*. **(B)** Relative mRNA expression of *CTB* via qRT-PCR. **(C)** Relative production of CTB protein via ELISA. Bars are color-coded by the co-infiltrated constructs: Black (CTB alone control), Blue (TIRCTB + TC1, TC12, or TC123), Orange (HIRCTB + HC1, HC12, or HC123), and Green (BIRCTB + BC1, BC12, or BC123). CTB in a single agroinfiltrated leaf was used as a control. The relative copy number, transcript (mRNA) levels, and protein production (CTB production) were all normalized to the CTB expression level measured at 3 DPI. **(D–F)** Phenotype of *Nicotiana benthamiana* leaves at 3 DPI. Leaves were agroinfiltrated with constructs derived from three geminiviral systems: TYLCV **(D)** HYVV **(E)** and BMCTV **(F)**. Mock: Infiltration with buffer only. CTB: Agroinfiltration expressing CTB alone. TIRCTB: Agroinfiltration expressing CTB harboring the IR of TYLCV. TIRCTB + TC1, TIRCTB + TC12, and TIRCTB + TC123: CTB co-expressed with TYLCV combination vectors described in the Figure 1 legend. HIRCTB: Agroinfiltration expressing CTB harboring IR of HYVV. HIRCTB + HC1, HIRCTB + HC12, and HIRCTB + HC123: CTB co-expressed with HYVV combination vectors described in the Figure 1 legend. BIRCTB: Agroinfiltration expressing CTB harboring the IR of BMCTV. BIRCTB + BC1, BIRCTB + BC12, and BIRCTB + BC123: CTB co-expressed with BMCTV combination vectors described in the Figure 1 legend. Data are presented as mean ± SE from three independent infiltrated samples. Significant differences were assessed using Dunnett’s one-way ANOVA. *p < 0.05; **p < 0.01; ***p < 0.001; ns, not significant.

We also tested whether episomal DNA amplification was associated with an increase in *CTB* copy number (Figure 2A). Real-time PCR was used to determine the *CTB* gene copy number in co-infiltrated *N. benthamiana* leaves with each replicon vector combination. Combined with TIRCTB and its replication-related genes, *CTB* gene amplification levels were high, ranging from 200- to 650-times compared to the *CTB* alone control. Similarly, the HIRCTB combination with its replication-related genes resulted in a comparable increase, achieving a 201- to 700-fold increase. The data indicated that the highest *CTB* amplification was achieved with TIRCTB + TC123 or HIRCTB + HC123, which was a 650- to 700-times increase compared to the *CTB* control. In contrast, the combination of BIRCTB and its replication-related genes showed only a 2-to 7-fold increase in *CTB* gene amplification. Co-infiltration with BIRCTB + BC1 resulted in the highest copy number (7-fold) of the *CTB* gene among the combinations of BIRCTB and BMCTV replication-related genes, which differed from the other cases (Figure 2A). In addition, we investigated the potential of various geminivirus-derived IR- and replication-related gene combinations to enhance *CTB* mRNA expression and protein production at 3 DPI. Total RNA and protein were extracted from *N. benthamiana* leaves collected at 3 DPI, and qRT-PCR and CTB protein quantities were measured using ELISA (Figures 2B,C). In the case of *CTB* mRNA expression, the three geminivirus combinations were highest in TIRCTB + TC123, HIRCTB + HC123, and BIRCTB + B1, and the relative expression was approximately 15-, 7-, and 10-times that of *CTB* alone (Figure 2B). Quantification of the CTB protein using ELISA revealed that, unlike the mRNA expression pattern, the highest expression was observed in BIRCTB + B1, showing an approximately 2.8-fold increase compared with *CTB* alone. The TIRCTB + TC123 and HIRCTB + HC123 combinations showed approximately 2.3-fold and 2-fold increases, respectively, compared to the *CTB* alone. Investigation of CTB expression and morphological changes according to each geminivirus combination showed that leaf necrosis was observed in the HIRCTB + HC123 combination. In other combinations, a change in the infiltrated spot to chlorosis was observed in *N. benthamiana* leaves.

Based on a previous study, when tGFP was expressed using the geminivirus system, the mRNA expression and tGFP fluorescence intensity increased proportionally with the increase in copy number. However, in the case of CTB expression, the highest CTB protein expression was observed in BMCTV rather than in TYLCV or HYVV, which had the highest copy number. This phenomenon is thought to be due to a reduction in mRNA and protein accumulation in the TYLCV or HYVV IR and Rep combination expressing CTB, resulting from severe leaf necrosis symptoms (Figures 2D–F).

### 3.3 Phenotypic changes resulting from transient *CTB* expression were observed in *Nicotiana benthamiana*

**FIGURE 3:**
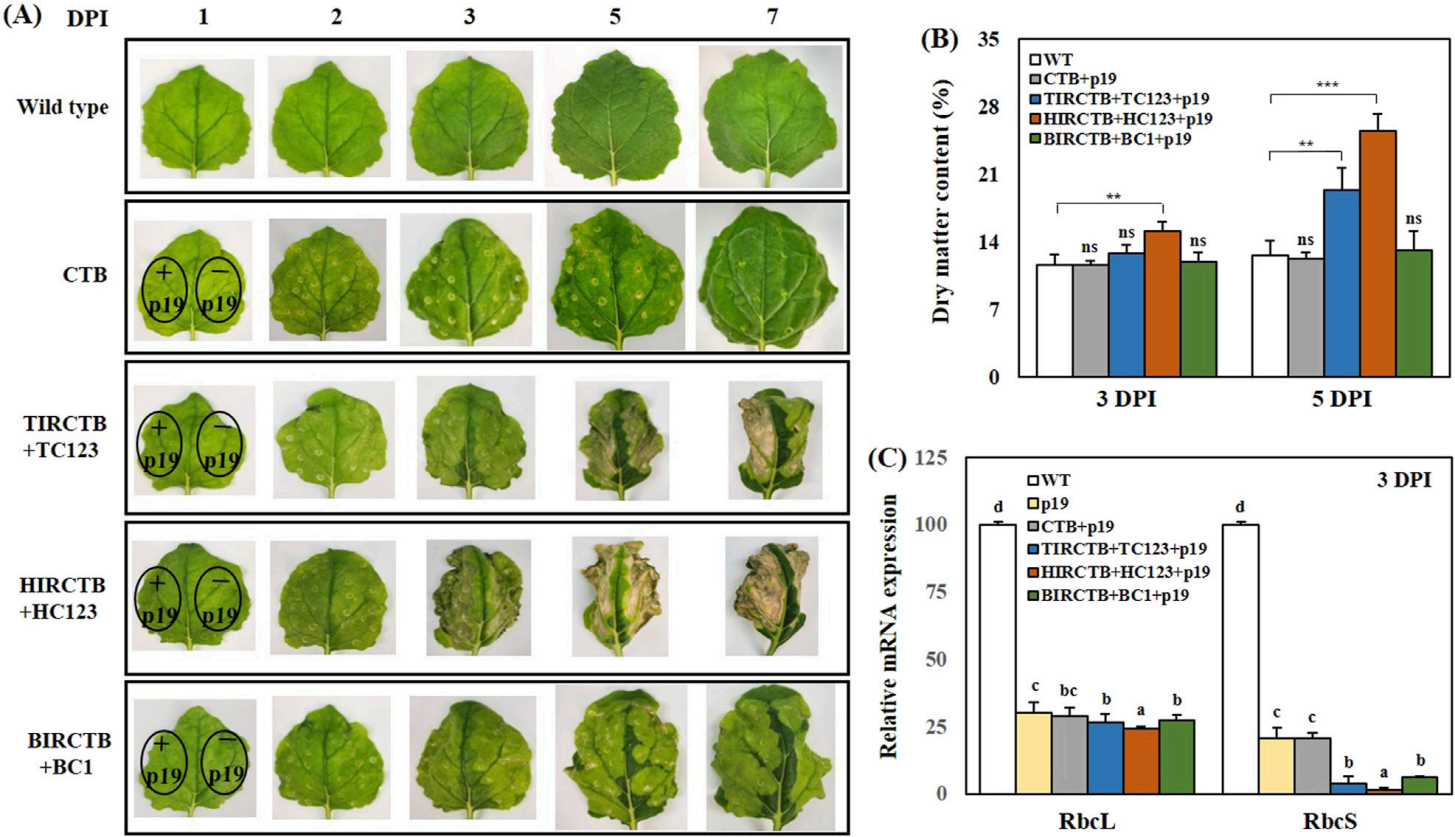
Typical phenotypes of agroinfiltrated *Nicotiana benthamiana* leaves. **(A)** Images show the visual appearance of leaves at 1, 2, 3, 5, and 7 DPI under visible light. The phenotypes are compared across leaves infiltrated with the CTB (alone) and co-infiltrated with three geminiviral vectors: TIRCTB + TC123, HIRCTB + HC123, and BIRCTB + BC1. All conditions were tested both with and without co-expression of the viral suppressor p19. **(B)** Phenotypic analysis of CTB transient expression at 3 and 5 DPI. **(C)** Relative mRNA expression of RuBisCO-related genes, as measured by qRT-PCR, at 3 DPI. *RbcL* and *RbcS* refer to the RuBisCO large and small subunits, respectively. WT: Wild-type leaves (untreated control). p19: Agroinfiltrated leaves expressing the p19 only. CTB + p19: Leaves co-infiltrated with CTB and p19. TIRCTB + TC123 + p19: Leaves co-infiltrated with TIRCTB, TC123, and p19. HIRCTB + HC123 + p19: Leaves co-infiltrated with HIRCTB, HC123, and p19. BIRCTB + BC1+p19: Leaves co-infiltrated with BIRCTB, BC1, and p19. Data are mean ± SE from three independent infiltrated samples. Significant differences were assessed using one-way ANOVA. Dunnett’s test was used for comparisons against a control group (∗∗*p* < 0.01; ∗∗∗*p* < 0.001; ns, not significant), while Tukey’s test was applied for all pairwise comparisons (*p* < 0.05). Groups were labelled with a compact letter display, where groups not sharing the same letter are statistically different.

We identified and selected three geminiviral vector combinations that were effective for high-level *CTB* expression: TIRCTB + TC123, HIRCTB + HC123, and BIRCTB + BC1. Subsequently, we investigated the time course of *CTB* gene expression and associated phenotypic alterations via co-agroinfiltration with p19. Following agroinfiltration, we noted a significant increase in leaf curling over time compared to the wild type (Figure 3A). and was severe in the HIRCTB + HC123 + p19 combination, visible to the naked eye as early as 2 DPI. This necrosis was directly correlated with a reduction in CTB antigen protein levels and activity. Furthermore, analysis of DMC at 3 and 5 DPI showed a 1.5- to 2-fold increase in the TIRCTB + TC123 + p19 and HIRCTB + HC123 + p19 combinations compared to CTB + p19 (used as the control), whereas the BIRCTB + BC1+p19 combination maintained DMC similar to that of CTB + p19 (Figure 3B).

To investigate the impact on the protein components of the photosystems, we performed qRT-PCR to analyze the expression patterns of *NbRbcL* and *NbRbcS*, the large and small subunits of the ribulose-1,5-bisphosphate carboxylase/oxygenase (RuBisCO) (Hamel et al., 2024b). The expression of genes involved in photosynthesis significantly decreased in the infiltrated leaves compared to the wild type at 3 DPI. This decline was pronounced from 3 DPI onwards in the HIRCTB + HC123 + p19 and TIRCTB + TC123 + p19 combinations (Figure 3C). These findings suggest that geminivirus-based vector combinations may impair the expression of chloroplast-related genes and contribute to ER stress induction and immune response activation, factors associated with the observed leaf necrosis. These critical observations led us to design and conduct subsequent experiments.

### 3.4 Geminivirus-mediated high CTB expression in *Nicotiana benthamiana* induces distinct ER stress and immune responses, leading to physiological decline

**FIGURE 4:**
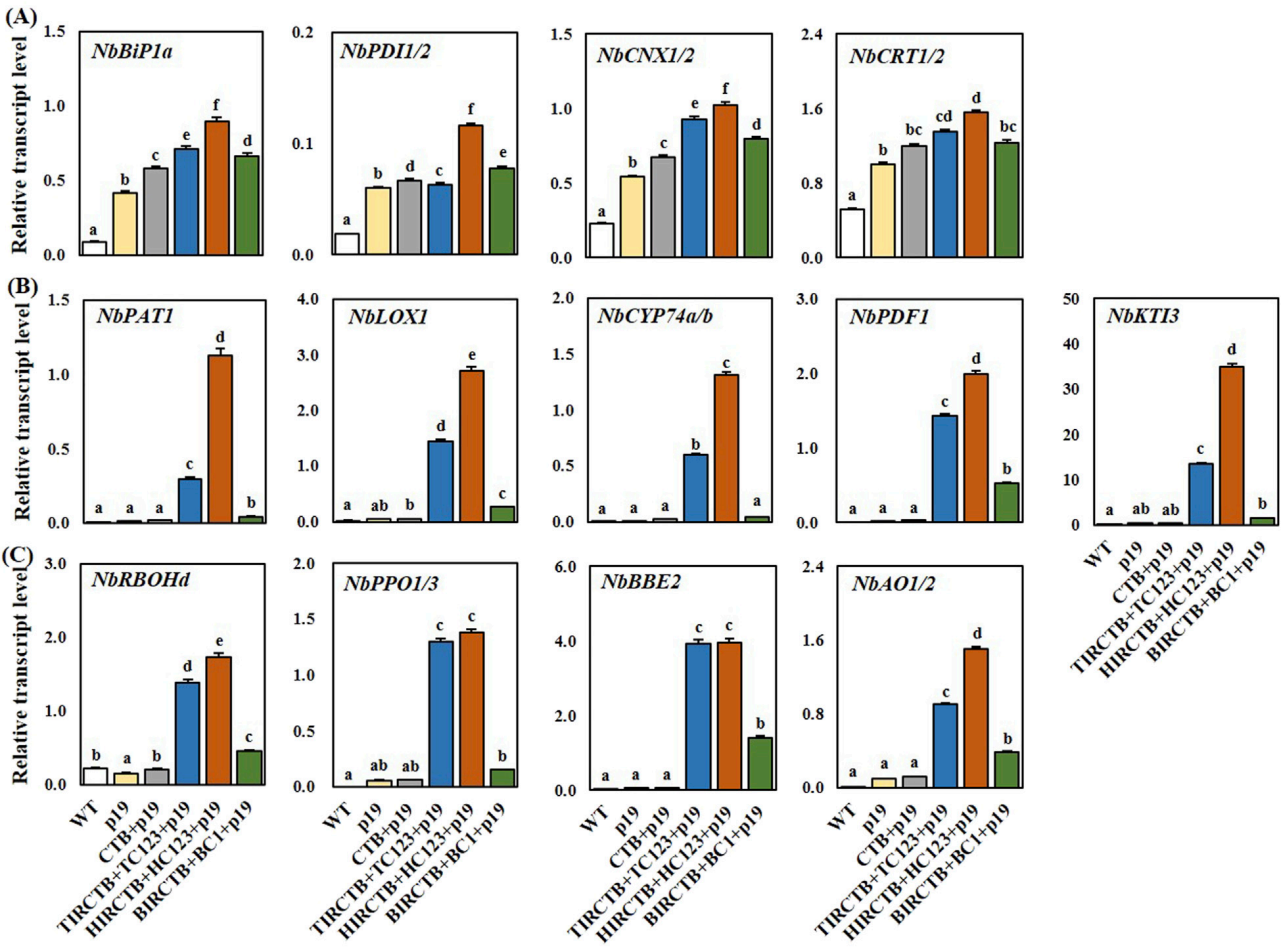
Relationship between stress response and CTB transient high expression using geminiviral-based vector systems in *Nicotiana benthamiana* at 3 DPI. Specifically: **(A)** UPR-related gene expression (*NbBiP1a, NbPDI1/2, NbCNX, and NbCRT*), **(B)** defense-related gene expression (*NbPAT1, NbLOX1, NbCYP74a/b, NbPDF1, and NbKTI3*), and **(C)** oxidative-related gene expression (*NbRBOHd, NbPPO1/3, NbBBE2, and NbAO1/2*) are shown. Total RNA was extracted from leaf extracted from leaf tissues at 3 DPI. The starting amounts of cDNA for each gene were normalized to that of actin RNA. WT: Wild-type leaves (untreated control). p19: Agroinfiltrated leaves expressing the p19 only. CTB + p19: Leaves co-infiltrated with CTB and p19. TIRCTB + TC123 + p19: Leaves co-infiltrated with TIRCTB, TC123, and p19. HIRCTB + HC123 + p19: Leaves co-infiltrated with HIRCTB, HC123, and p19. BIRCTB + BC1+p19: Leaves co-infiltrated with BIRCTB, BC1, and p19. Data are mean ± SE from three independent infiltrated samples. Statistical analysis was performed using one-way ANOVA followed by Tukey’s test (*p* < 0.05). Groups were labelled with a compact letter display, where groups not sharing the same letter are statistically different.

**FIGURE 5:**
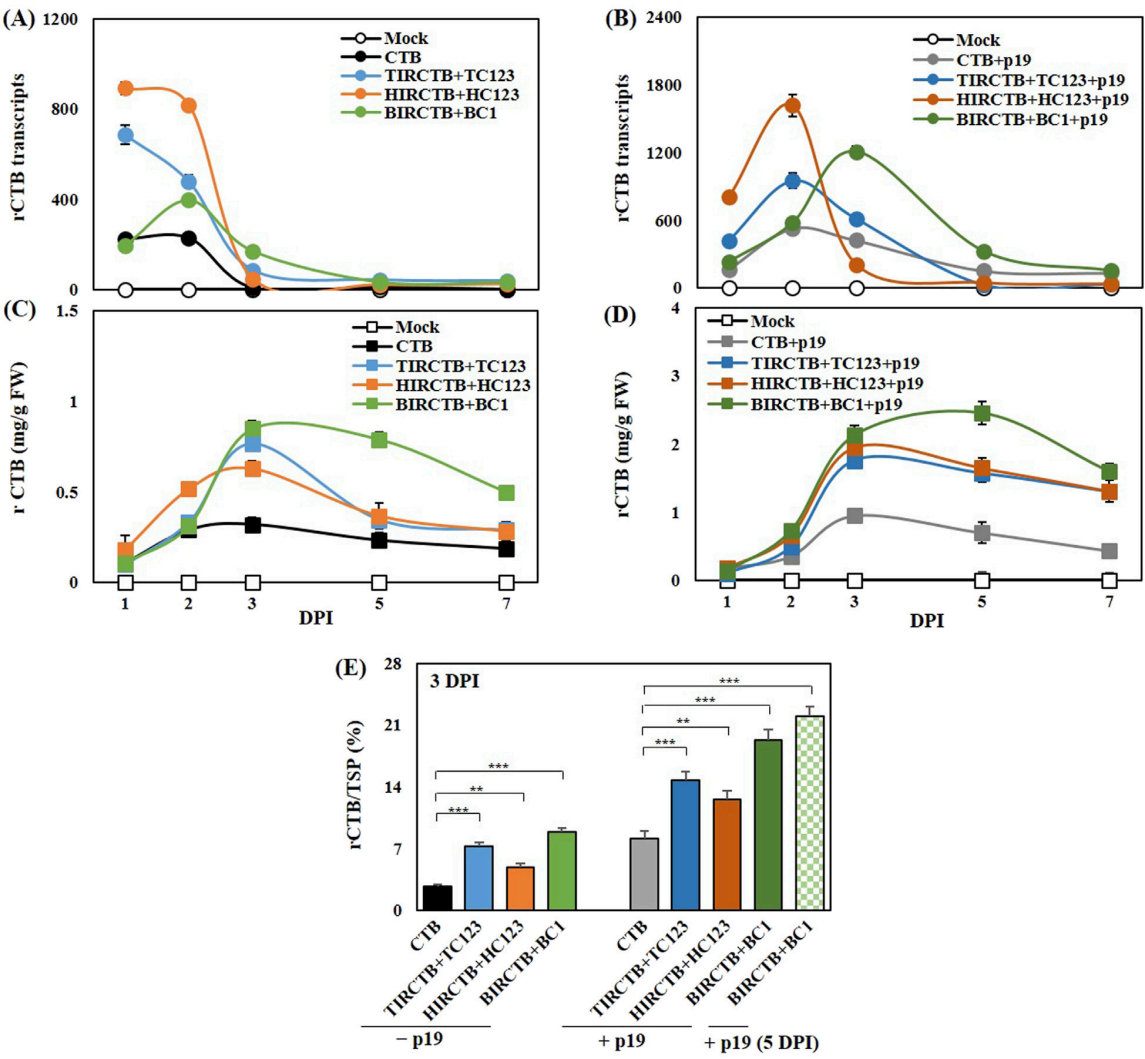
Effects of the p19 silencing suppressor on *CTB* mRNA and protein accumulation in agroinfiltrated *Nicotiana benthamiana* leaves. The results are compared across leaves infiltrated with the CTB (alone) and co-infiltrated with three geminiviral vectors: TIRCTB + TC123, HIRCTB + HC123, and BIRCTB + BC1. All infiltration conditions were tested both with and without co-expression of p19 at 1, 2, 3, 5, and 7 DPI. The mock is a leaf infiltrated with buffer only. **(A,B)** Time-dependent CTB mRNA expression is compared in samples collected at various time points, without and with p19 co-expression. **(C,D)** CTB protein production, quantified via ELISA, is compared at various time points under the same conditions as **(A,B)**. **(E)** Comparison of CTB protein accumulation in total soluble proteins at 3 DPI and 5 DPI (only BIRCTB + BC1+p19 sample). Data are mean ± SE from three independent infiltrated samples. Significant differences were assessed using Dunnett’s one-way ANOVA, **p < 0.01; ***p < 0.001.

We believe that maintaining physiological activity and securing biomass are crucial when aiming for high foreign gene expression using the geminivirus vector system; therefore; we analyzed the underlying causes. We observed severe leaf necrosis, chlorosis, and leaf curling over time in the two optimized geminiviral vector combinations designed for high CTB expression accumulated in the ER (Figure 5). The most pronounced symptoms were observed with the TIRCTB + TC123 + p19 and HIRCTB + HC123 + p19 optimal combinations from 3 DPI. In contrast, when CTB + p19 and BIRCTB + BC1+p19 were expressed, leaf curling was observed, but no decrease in FW due to leaf necrosis was observed. These findings align with previous reports (Hamorsky et al., 2015), which indicated that whereas glycosylated CTB targeted to the ER resulted in milder leaf necrosis, CTB with a modified glycosylation pattern led to severe leaf necrosis and reduced expression levels. To understand the cause of the decreased recombinant protein expression and reduced biomass observed through transient expression-induced leaf necrosis and chlorosis, we performed qRT-PCR on a selection of genes based on reports concerning gene expression changes during transient recombinant protein expression in *N. benthamiana* (Hamel et al., 2024a; 2024b). We specifically examined the expression patterns of UPR-related genes involved in ER stress due to the accumulation of high levels of proteins in the ER, as well as genes involved in the immune response at 3 DPI. We performed qRT-PCR on a comprehensive set of genes, including those related to ER stress (Figure 4): *NbBiP1a, NbPDI1/2, NbCNX1/2,* and *NbCRT1/2*. We also analyzed the defense genes *NbPAT1, NbLOX1, NbCYP74a/b, NbPDF1, NbKTI3*, and oxidative stress-related genes *NbRBOHd, NbPPO1/3, NbBBE2, and NbAO1/2*. The expression of UPR-related genes showed a similar pattern across all three geminivirus combinations at 3 DPI, with the HIRCTB + HC123 + p19 combination exhibiting the highest expression levels among them (Figure 4A). The expression of genes involved in immune responses increased at 3 DPI, with a notable surge in the TIRCTB + TC123 + p19 and HIRCTB + HC123 + p19 combinations. In contrast, when p19 alone, CTB + p19 and BIRCTB + BC1+p19 were expressed, the activity of immune response genes remained low (Figures 4B,C).

### 3.5 Enhanced CTB expression via geminivirus-based vectors and p19-mediated post-transcriptional gene silencing (PTGS) suppression in *Nicotiana benthamiana*

Following a series of experiments, TIRCTB + TC123, HIRCTB + HC123, and BIRCTB + BC1 emerged as the optimal pairings of geminivirus-based IR and replication-related genes for robust CTB expression. A known limitation of transient expression in tobacco, despite strong viral vector-induced expression, is the rapid mRNA degradation caused by PTGS, which severely impacts target protein yields (Garabagi et al., 2012). Consistent with prior studies, we incorporated p19, a potent PTGS suppressor, to address this. We diluted p19 at a 2:1 ratio with the selected optimal CTB-expressing geminiviral vector combinations and collected samples at 1, 2, 3, 5, and 7 DPI. Total RNA was extracted, and *CTB* mRNA expression was assessed. The combinations without p19 showed maximum expression at 1 DPI, except for HIRCTB + HC123, which peaked on 2 DPI. In contrast, co-expression with p19 resulted in peak levels at 2 or 3 DPI across all combinations, demonstrating a 1.7- to 2.8-fold increase compared to the control vector expressing CTB alone (Figures 5A,B).

For the quantification of CTB over time, BIRCTB + BC1+p19 showed maximum production and high content at 5 DPI, reaching 2.5 mg/g FW and 2.2% of the total protein. In the case of TIRCTB + TC123 + p19 and HIRCTB + C123 + p19, high production and content were observed at 3 DPI, with values of 2.0 mg/g FW, 15%, and 1.8 mg/g FW, 13%, respectively (Figures 5C,D). Analysis of the proportion of CTB protein in the total protein revealed a maximum content of 22% at 5 DPI in the BMCTV virus element combination when co-expressed with p19 (Figure 5E). These results contrast with our previous findings, in which an increase in replicon copy number correlated with increased tGFP expression. Specifically, for CTB expression, HIRCTB + HC123 + p19 showed the highest replicon copy number, but its expression level was low. We hypothesized that this discrepancy was due to ER stress induced by early overexpression in HIRCTB + HC123 + p19 cells, leading to improper protein folding. This is thought to have triggered an immune response via the activation of UPR gene expression and the ERAD system (Hamorsky et al., 2015; Hamel et al., 2024a; 2024b).

### 3.6 Successful assembly and GM_1_-binding of recombinant CTB in geminivirus-based *Nicotiana benthamiana* for molecular farming

**FIGURE 6:**
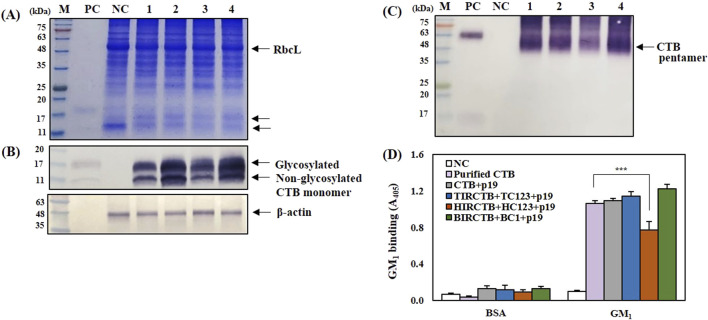
Analysis of CTB protein production and activity in agroinfiltrated *Nicotiana benthamiana* leaves co-expressed with p19 at 3 DPI. **(A)** SDS-PAGE gel stained to show total soluble proteins. **(B)** and **(C)** Western blot analysis of CTB protein using an anti-CTB antibody. β-actin was used as an internal loading control. **(D)** GM_1_ ELISA analysis showing CTB pentamer formation. The binding affinity of the plant-produced CTB to the GM_1_ receptor was measured to assess activity. Lane M: Prestained protein marker. Lane PC: Purified HEK293-derived CTB protein (Positive Control). Lane NC: Leaf extracts infiltrated with buffer only (Negative Control). Lane 1: Leaf extracts from co-infiltration with CTB + p19. Lane 2: Leaf extracts from co-infiltration with TIRCTB + TC123 + p19. Lane 3: Leaf extracts from co-infiltration with HIRCTB + HC123 + p19. Lane 4: Leaf extracts from co-infiltration with BIRCTB + BC1+p19. Data are mean ± SE from three independent infiltrated samples. Significant differences were assessed using Dunnett’s one-way ANOVA (***p < 0.001).

To confirm the formation of monomeric and pentameric forms of the CTB antigen protein and its binding to GM_1_-ganglioside in the CTB + p19, TIRCTB + TC123 + p19, HIRCTB + HC123 + p19, and BIRCTB + BC1+p19 combinations, SDS-PAGE, Western blotting, and GM_1_-ELISA were performed. Using 25 μg of leaf protein, SDS-PAGE and Western blot analysis revealed bands of similar size to the glycosylated (11–17 kDa) and non-glycosylated CTB derived from animal cells, which were used as controls. These bands were confirmed on a reducing SDS-PAGE gel with a CTB-specific antibody (Figures 6A,B). Furthermore, under non-reducing conditions, CTB produced in the three geminivirus vector combinations was confirmed to form pentamers of 55–63 kDa, similar to the commercial purified CTB positive control (Figure 6C). In agreement with previous studies, the expression of CTB protein with a GluB-1 signal peptide and KDEL sequence at the N- and C-terminus in rice was *N*-glycosylated at Asn32 with immunoactive glycan structures against anti-horse radish peroxidase antibody, but some of the CTB was not glycosylated (Kajiura et al., 2013). The GM_1_-ELISA results, which assessed the binding affinity to GM_1_-ganglioside, confirmed that the plant-derived CTB antigen protein expressed at 3 DPI, exhibited an activity similar to that of the positive control CTB. However, the HIRCTB + HC123 + p19 combination resulted in an approximately 25% reduction in GM_1_-ganglioside binding ability compared to the positive control (Figure 6D).

## 4 Discussion

Transient expression using agroinfiltration is widely used to produce recombinant proteins within days. Recent research has focused on developing various expression vectors that use genetic elements from plant viruses, using diverse strategies to achieve high expression levels of the gene of interest. Several advanced viral vector systems have been developed to enhance the transient expression yield. The magnICON® system, a deconstructed tobamovirus-based vector system, uses the Magnifection method for viral replicon delivery, which results in rapid GFP protein accumulation of 2.5 to 4 g/kg FW within a week (Marillonnet et al., 2005). Similarly, the highly effective TRBO system, a modified TMV-based vector, replaces non-essential viral genes with genes of interest. This approach boosts agroinfection efficiency and increases GFP protein expression to 3.3–5.5 g/kg FW (Lindbo, 2007). Although the BeYDV-based system initially produced lower yields, it underwent significant optimization to become a highly versatile platform. Modifications such as a double terminator system and a single nucleotide change in the 5′-UTR have dramatically increased protein yields, with some studies reporting 3–5 g/kg FW for GFP, which constitutes approximately 50% of the TSP (Diamos and Mason, 2018), and 2 mg/g for Norwalk virus capsid protein (Diamos and Mason, 2019). A key advantage of the BeYDV system is its ability to co-express multiple large gene fragments simultaneously, making it ideal for producing complex multi-subunit proteins. For example, it has been used to produce monoclonal antibodies, such as anti-Zika (Diamos et al., 2020b), anti-CTLA-4 (Bulaon et al., 2023), and West Nile Virus chimeric virus-like particles (Chen et al., 2011). The choice of viral vector is crucial and depends on the specific application. TMV-based systems, such as TRBO, are highly efficient in *N. benthamiana* but have limited host range and gene co-expression capabilities (Hoshikawa et al., 2019; Lai et al., 2012). In contrast, the BeYDV system is more versatile, supporting multiple gene expressions across various dicot plants, although it requires a higher concentration of Agrobacterium for infiltration (Diamos and Mason, 2018). The continuous development of these deconstructed viral vector platforms through innovative genetic modifications underscores their essential role in advancing plant-based protein production for both research and commercial applications.

Here, we successfully developed and optimized a geminivirus-derived deconstructed vector system for the high-level transient expression of *CTB* in *N. benthamiana* leaves. Building upon our previous work demonstrating efficient tGFP expression (Kim et al., 2024), we focused on enhancing the production of biologically functional CTB, a highly relevant recombinant protein for vaccine development. In agreement with previous studies, we observed robust episomal replicon formation in all co-infiltrated *N. benthamiana* leaves in this study. As shown in Figure 2, the CTB gene copy number was significantly amplified by the TIRCTB + TC123 and HIRCTB + HC123 combinations, achieving a 650- to 700-fold increase over that of the negative control. The BIRCTB + BC1 combination exhibited only a modest 2–7-fold increase (Figure 2). This differential replication underscores the varying efficiencies of different geminiviral elements in driving the formation of replicons. Interestingly, whereas mRNA expression correlated with high copy numbers in the TIRCTB + TC123 and HIRCTB + HC123 combinations, CTB protein production showed a different pattern. The BIRCTB + BC1 combination, despite having lower DNA amplification, exhibited the highest CTB protein yield (approximately 2.8-fold increase), surpassing those of TIRCTB + TC123 (2.3-fold) and HIRCTB + HC123 (2-fold). This discrepancy suggests that an increase in replicon copy number may not always lead to a proportional increase in mRNA transcription and protein accumulation in the geminiviral vector system. A notable observation was the onset of leaf necrosis, chlorosis, and curling in *N. benthamiana* leaves expressing CTB, particularly severe in TYLCV (TIRCTB + TC123) and HYVV (HIRCTB + HC123), where DNA amplification was highest. We hypothesize that infiltrated leaf necrosis is directly associated with the accumulation of CTB protein levels and activity.

As noted by Huang et al. (2009), hepatitis B core antigen (HBc) was produced at 0.8 mg/g FW with the BeYDV-derived vector system, but the necrosis triggered by the pBYHBc/REP110/P19 combination at 5 DPI. This study further showed that no necrotic phenotypes were observed in leaves infiltrated with pBYGFP.R or the pBYGFP/REP110/P19 combination, suggesting that necrosis is linked to the HBc recombinant protein rather than the Rep/RepA proteins. Consequently, we propose that an optimal balance of Rep/RepA is crucial for each specific target protein to maximize its expression potential without exacerbating negative effects. Furthermore, Diamos and Mason (2019) suggested that carefully reducing Rep/RepA expression can alleviate cell death from geminiviral replicons, leading to minimal yield reduction for non-toxic proteins such as GFP, but increased accumulation of toxic proteins such as Norwalk virus capsid protein. Reducing the hypersensitive response allows for greater protein accumulation from genes that would otherwise be limited by the cell death. In addition, because the hypersensitive response and RNA silencing are linked, alleviating one may also prevent premature gene silencing of BeYDV-based vectors (Zvereva and Pooggin, 2012).

We show that phenotypic symptoms and gene expression changes indicate that the high-level accumulation of foreign protein, particularly when targeted to the ER, can induce plant stress responses. The HIRCTB + HC123 + p19 combination exhibited a decrease in DMC and a dramatic decline in the expression of *RuBisCO* genes (*RbcL* and *RbcS*) from 1 DPI, suggesting a significant impairment of plant physiological activity (Figure 3). According to a recent report, host-plant responses to foreign protein expression not only provide the research community with a useful set of marker genes for studying ER stress associated with UPR and plant immune response genes in *N. benthamiana*, but also highlight its importance as a model organism for molecular farming and plant immunity studies (Ranawaka et al., 2023; Hamel et al., 2024a; 2024b). Understanding the impact of these stresses is crucial for ensuring the sustainability of molecular farming. This is especially true because proteins of biopharmaceutical interest often require precise folding and complex post-translational modifications, such as sophisticated glycosylation patterns or assembly into specific quaternary structures. Overloading the host cell’s ER with these complex proteins can lead to ER stress and activate the UPR. If this stress is not resolved, it can severely compromise plant cell health and viability. This results in poor biomass quality and insufficient protein yields at harvest. Given that geminivirus-based transient production of recombinant proteins in *N. benthamiana* is the most progressive, information leading to the mechanism of failed UPR, ERAD, and PCD can lead to healthier plant conditions and increased protein accumulation. Our qRT-PCR analysis (Figure 5) revealed an upregulation of UPR-related genes (*NbBiP1a*, *NbPDI1/2*, *NbCNX*, and *NbCRT*) and immune response genes (*NbPAT1*, *NbLOX1*, *NbCYP74a/b*, *NbPDF1*, and *NbKTI3*). This effect was most pronounced in the TYLCV and HYVV combinations. This aligns with previous reports indicating that ER stress and immune activation can negatively impact recombinant protein accumulation and host fitness (Hamorsky et al., 2015; Hamel et al., 2024a; 2024b). The severe symptoms observed in HIRCTB + HC123 + p19 and TIRCTB + TC123 + p19 combinations likely explain the reduced protein accumulation despite high gene copy numbers, as the plant’s resources are diverted to stress management or tissue damage, limiting the overall biosynthetic capacity. In contrast, milder symptoms and sustained physiological activity in the BIRCTB + BC1+p19 combination likely contributed to its superior protein yield despite lower initial gene amplification.

PTGS is a significant hurdle in achieving high-level recombinant protein expression in *N. benthamiana*. As previously shown with tGFP, our study also demonstrated that p19, a well-known PTGS suppressor, effectively enhanced CTB mRNA and protein accumulation. (Kim et al., 2024; Lee et al., 2024). Co-infiltration with p19 shifted the peak mRNA expression from 1 DPI (without p19) to 2 or 3 DPI, resulting in a 1.7- to 2.8-fold increase in the CTB mRNA levels. Crucially, p19 co-expression led to a maximum CTB protein production of 2.5 mg/g FW (2.2% of total protein) at 5 DPI in the BIRCTB + BC1 combination, and high levels (2.0 mg/g FW, 15% in TIR-CTB + TC123; 1.8 mg/g FW, 13% in HIRCTB + C123) at 3 DPI. This highlights p19’s critical role in stabilizing mRNA and allowing for prolonged protein synthesis, overcoming the rapid degradation characteristic of transient expression systems. However, co-expression with p19 still led to approximately 25% reduction in GM_1_-ganglioside binding ability in the HIRCTB + HC123 + p19 combination, suggesting potential intricate interactions or additional stress factors at play, even with gene silencing. Despite the observed physiological stress and variable expression levels, the plant-derived CTB produced by all three optimized geminivirus vector combinations (TIRCTB + TC123, HIRCTB + HC123, and BIRCTB + BC1) with p19 successfully formed both monomeric (11–17 kDa) and pentameric (55–63 kDa) structures, consistent with the control CTB from animal cells. GM_1_-ELISA results confirmed that plant-derived CTB exhibited a binding affinity to GM_1_-ganglioside as the standard CTB, indicating its biological functionality. This demonstrates the potential of *N. benthamiana* as a viable platform for producing complex, functionally active recombinant proteins, such as CTB. While this study demonstrates significant progress in plant-based CTB production, future research should focus on mitigating the observed plant stress responses, particularly leaf necrosis, to further enhance the overall protein yield and biomass. Strategies to achieve this could include optimizing promoter strength, exploring alternative subcellular targeting, or using targeted glycoengineering to improve protein folding and reduce ER stress.

Previous study (Kim et al., 2024) and this study had limited data, necessitating a parallel comparison among results using three viral vectors to express recombinant proteins. However, comparing these results, the HYVV-based viral vector appeared to induce more necrosis than other viral vectors (Figure 3A), while not showing higher recombinant protein levels (Figure 5; Kim et al., 2024). Therefore, the HYVV-based viral vector was considered to have little particular advantage, and experiments using it were discontinued. Recombinant protein expression is now being performed using BMCTV- and TYLCV-based viral vectors, respectively. When testing the expression of recombinant proteins, including bovine growth factors, using TYLCV- and BMCTV-based viral vectors, BMCTV-based viral vector was preferable in the case of TYLCV-based viral vector induced necrosis in agroinfiltrated leaves (Unpublished data). Whereas when TYLCV-based viral vector did not induce necrosis, the use of TYLCV-based viral vector was considered to be more advantageous (Kim et al., 2024; Unpublished data).

## 5 Conclusion

In this study, we successfully established an optimized geminivirus-derived deconstructed vector system for the high-level transient expression of functional CTB in *N. benthamiana*. We demonstrated that whereas high DNA amplification is crucial, the ultimate protein yield is influenced by host-plant health and stress responses. Despite its lower amplification rates, the BMCTV-based system proved to be the most effective for CTB production because it maintained a healthy host environment. These results provide valuable guidance for the future development of deconstructed geminiviral vectors, emphasizing the need to engineer systems that achieve a stable, subthreshold level of expression that maximizes biomanufacturing output without triggering detrimental host responses. Our findings highlight that severe plant physiological stress, characterized by necrosis and upregulation of UPR and immune-related genes, can counteract the benefits of high gene copy numbers. The critical role of p19 in mitigating gene silencing and enhancing gene expression was confirmed. Crucially, the plant-produced CTB exhibited correct structural assembly and biological functionality, affirming the potential of *N. benthamiana* as a valuable platform for recombinant protein production. To optimize geminiviral vector systems for applications such as biopharmaceutical protein production and gene editing, it is crucial to understand these factors. This will help minimize unwanted cell death and maximize the desired outcomes. Future efforts should focus on strategies to reduce plant stress, allowing us to achieve the high-yield potential of this promising plant-based molecular farming.

## Data Availability

The datasets presented in this study can be found in online repositories. The names of the repository/repositories and accession number(s) can be found in the article/Supplementary Material.
