# Supplementary material for: Efficient production of functional cholera toxin B subunit using geminiviral replicons in Nicotiana benthamiana
Source: Front Bioeng Biotechnol. 2025 Nov 14;13:1693569. doi: 10.3389/fbioe.2025.1693569 (PMC12660276; doi:10.3389/fbioe.2025.1693569)
Supplement: Supplementary file 1 [file Supplementaryfile1.docx]

**Supplementary Figure 1.** Conformation of IR-carrying CTB replicons in co-infiltrated *N. benthamiana* leaves with different vector combinations. (A) Outward-facing primer design, which can be only amplified using replicon DNA as a template. (B) Genomic PCR products using outward-facing primers. Lane M: 1 kb DNA ladder, Lane 1: CTB only, Lanes 2–4: co-infiltrated leaf with TIRCTB+TC1, TC12 or TC123, Lanes 5–7: co-infiltrated leaf with HIRCTB+HC1, HC12 or HC123, Lanes 8–10: co-infiltrated leaf with BIRCTB+BC1, BC12 or BC123. Black arrows represent primer binding sites for the polymerase chain reaction.

**
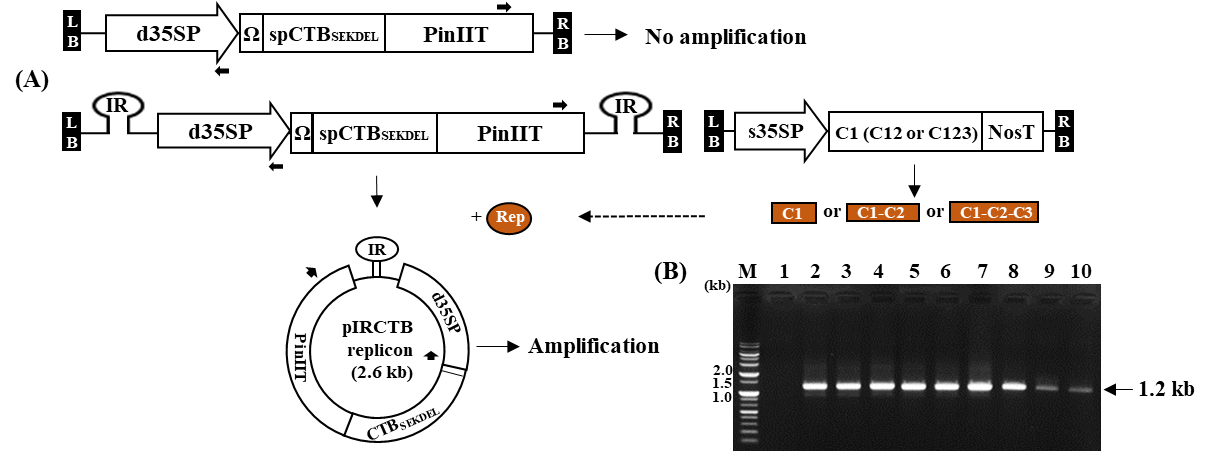
**
