# Supplementary material for: Efficient production of functional cholera toxin B subunit using geminiviral replicons in Nicotiana benthamiana
Source: Front Bioeng Biotechnol. 2025 Nov 14;13:1693569. doi: 10.3389/fbioe.2025.1693569 (PMC12660276; doi:10.3389/fbioe.2025.1693569)
Supplement: Supplementary file 2 [file Table1.docx]

| **Gene** | **Genbank**  **accession no.** | **Primer name** | **DNA sequence (5′–3′)** | **Purpose** |
| --- | --- | --- | --- | --- |
| *TYLCV* | KF225312 | TYLCV IR F  TYLCV IR R | TTGAAGACATCTCAGGAGTTGAAATGAATCGGTGTCCCT  TTGAAGACAACTCGAGTATTGCAAGACAAAAAACTTGGG | IR cloning |
| *HYVV* | GQ477135 | HYVV IR F  HYVV IR R | TTGAAGACATCTCAGGAGTTGACTTGGTCAATTGGG  TTGAAGACAACTCGAGTACTTACACCGTTAAAATTAGGGCTGA |  |
| *BMCTV* | U56975 | BMCTV IR F  BMCTV IR R | TTGAAGACATCTCAGGAGGTTACTATTCCTATTGGGGGCTCT  TTGAAGACAACTCGAGTATTATAAATACCTATACGTATTCGTATAGC |  |
| *Not applicable* | Not applicable | Replicon PT F1  Replicon d35S R2 | TGTGGCCTTAATTGAATCATC  AGAGGAAGGGTCTTGCGAAG | Episomal  replicon  DNA PCR |
| *NbAct-b* | JQ256516 | β-actin F1  β-actin R1 | CCACCGGTATTGTGTTGGAC  CCTGACAATTTCCCGCTCAG | qRT-PCR |
| *CTB* | MG356518 | CTB F2  CTB R2 | CCAGGTAGTCAGCACATCGA  TGTTAGCCATTGAGATTGCGG |  |

**Supplementary Table S1.** List of primers used in this study.
