# Supplementary material for: Efficient production of functional cholera toxin B subunit using geminiviral replicons in Nicotiana benthamiana
Source: Front Bioeng Biotechnol. 2025 Nov 14;13:1693569. doi: 10.3389/fbioe.2025.1693569 (PMC12660276; doi:10.3389/fbioe.2025.1693569)
Supplement: Supplementary file 3 [file Table2.docx]

**Supplementary Table S2.** List of genes and primers for qRT-PCR.

| **Gene type** | **Gene targeted** | **Gene identification no. (Niben)** | **Primer name** | **DNA sequence (5′–3′)** |
| --- | --- | --- | --- | --- |
| Reference gene | *NbAct-b* | JQ256516 | β-actin F1  β-actin R1 | CCACCGGTATTGTGTTGGAC  CCTGACAATTTCCCGCTCAG |
| Chloroplast-related^a^ | *NbRbcL* | Niben101Scf00173g09004 | NbRbcL F1  NbRbcL R1 | GGCTTTGTTGATTTACTGCG  GTCCTAAAGTTCCTCCACCG |
|  | *NbRbcS* | Niben101Scf02381g04022 | NbRbcS F1  NbRbcS R1 | GCAGATACTGGACGATGTGG  GTAGGCGATGAAACTGATGC |
| UPR-related^b^ | *NbBiP1a* | Niben101Scf08590g00005 | NbBiP1a F1  NbBiP1a R1 | GAAACAGCCTGGAGACTTACG  CATAATCCTCCTTCTCAGCG |
|  | *NbPDI1/2* | Niben101Scf05405g08004  Niben101Scf02827g08001 | NbPDI1/2 F1  NbPDI1/2 R1 | GTACTTTGGTATTGGCGGTG  AGGGCTTTAGGTTGTCTTCC |
|  | *NbCNX1/2* | Niben101Scf03777g00002  Niben101Scf01596g12004 | NbCNX1/2 F1  NbCNX1/2 R1 | GGTTGATGAGGAAGATGGTG  GATTTGGAATATCCCTTGGC |
|  | *NbCRT1/2* | Niben101Scf00466g04036  Niben101Scf00332g06011 | NbCRT1/2 F1  NbCRT1/2 R1 | GCTTGTACTCTGACTGGGACC  TCACCATCTTCTTCATCATCC |
| Defense-related^b^ | *NbPAT1* | Niben101Scf06277g00007.1 | NbPAT1 F1  NbPAT1 R1 | AGAAGCCAGTGTCAAGGGTC  GAAAGCAACTGAGCAAAGTGG |
|  | *NbLOX1* | Niben101Scf01434g03006 | NbLOX1 F1  NbLOX1 R1 | GATGAATGGTGATGAGAAATGG  ATTCCCTTGCCAGTAAGTCC |
|  | *NbCYP74a/b* | Niben101Scf04787g02002  Niben101Scf07709g00001 | NbCYP74a/b F1  NbCYP74a/b R1 | GTAAATGGCTTTTTCCTCAGC  CTGTAATCACTTTTCACGAGACC |
|  | *NbPDF1* | Niben101Scf17290g01005 | NbPDF1 F1  NbPDF1 R1 | TCTCCTCTTGTGCTTCCTCC  TGCTCCCTCTTTCTCTTTGC |
|  | *NbKTI3* | Niben101Scf06424g00003 | NbKTI3 F1  NbKTI3 R1 | GAACCCTAACGAAAATATCATCC  TCTACTCCACCAACTGTCACC |
|  | *NbRBOHd* | Niben101Scf02581g04013 | NbRBOHd F1  NbRBOHd R1 | TGCTAAACCTAATTGGCGTAAT  GGCGTGTTGTCTTAGTTCTTTG |
|  | *NbPPO1/3* | Niben101Scf04384g02014  Niben101Scf00180g08002 | NbPPO1/3 F1  NbPPO1/3 R1 | TTTAGTGGTTGAAGGGATAGAGC  GCAAACTCAGCATTATTCGG |
|  | *NbBBE2* | Niben101Scf00944g01001 | NbBBE2 F1  NbBBE2 R1 | GGAGGTCTGGAAGGATTATGG  GAATGGGATAGCAGATGTTGC |
|  | *NbAO1/2* | Niben101Scf03026g01009  Niben101Scf22432g00001 | NbAO1/2 F1  NbAO1/2 R1 | ACCTTGTGAACCCAATTATGAAG  TGTGACTCAATATGGCAATGG |

This study provides the gene type, gene identification number (Niben), primer name, and sequence for each qRT-PCR primer set. The identification numbers for chloroplast-related, UPR-related, and defense-related genes from *N. benthamiana* are available on the Solanaceae Genomics Network (SGN; https://solgenomics.net/). The ^a^Primers and ^b^Primers were obtained from Hamel et al., 2024a and 2024b, respectively.
